# Observation and analysis of diving beetle movements while swimming

**DOI:** 10.1038/s41598-021-96158-1

**Published:** 2021-08-16

**Authors:** Debo Qi, Chengchun Zhang, Jingwei He, Yongli Yue, Jing Wang, Dunhui Xiao

**Affiliations:** 1 Key Laboratory of Bionic Engineering (Ministry of Education), Jilin University, Room 218, Bionics Building, 5988# Renmin Street, Changchun, 130025 China; 2 State Key Laboratory of Automotive Simulation and Control, Jilin University, Room 218, Bionics Building, 5988# Renmin Street, Changchun, 130025 China; 3 Weihai Institute for Bionics, Jilin University, Weihai, 264402 China; 4 College of Physics, Jilin University, Changchun, 130012 China; 5 ZCCE, College of Engineering, Swansea University, Swansea, SA1 8EN UK

**Keywords:** Behavioural methods, Biomimetics

## Abstract

The fast swimming speed, flexible cornering, and high propulsion efficiency of diving beetles are primarily achieved by their two powerful hind legs. Unlike other aquatic organisms, such as turtle, jellyfish, fish and frog et al., the diving beetle could complete retreating motion without turning around, and the turning radius is small for this kind of propulsion mode. However, most bionic vehicles have not contained these advantages, the study about this propulsion method is useful for the design of bionic robots. In this paper, the swimming videos of the diving beetle, including forwarding, turning and retreating, were captured by two synchronized high-speed cameras, and were analyzed via SIMI Motion. The analysis results revealed that the swimming speed initially increased quickly to a maximum at 60% of the power stroke, and then decreased. During the power stroke, the diving beetle stretched its tibias and tarsi, the bristles on both sides of which were shaped like paddles, to maximize the cross-sectional areas against the water to achieve the maximum thrust. During the recovery stroke, the diving beetle rotated its tarsi and folded the bristles to minimize the cross-sectional areas to reduce the drag force. For one turning motion (turn right about 90 degrees), it takes only one motion cycle for the diving beetle to complete it. During the retreating motion, the average acceleration was close to 9.8 m/s^2^ in the first 25 ms. Finally, based on the diving beetle's hind-leg movement pattern, a kinematic model was constructed, and according to this model and the motion data of the joint angles, the motion trajectories of the hind legs were obtained by using MATLAB. Since the advantages of this propulsion method, it may become a new bionic propulsion method, and the motion data and kinematic model of the hind legs will be helpful in the design of bionic underwater unmanned vehicles.

## Introduction

Diving beetles are aquatic organisms with the capabilities of flying in air, crawling on land, and swimming in water. In particular, the excellent maneuverability of diving beetles in water, including their rapid propulsion, flexible turning, and rapid retreating, have attracted extensive research interest^1–3^.

Diving beetles can swim rapidly due to their streamlined body shape, two flattened hind legs, and special mode of marine propulsion. They are good swimmers^4–7^. Compared with other aquatic organisms, such as turtle^8^, jellyfish^9^, fish^10,11^ and frog^12^, the diving beetles are more flexible, for which they could complete retreating motion without turning around, and the turning radius is small for this kind of propulsion mode^13^, whereas the mentioned creatures can usually move in only one direction and have a larger turning radius. Their streamlined body shapes contribute to the reduction of the resistance of water and the improvement of stability while swimming^14^. As the major propulsion organ, the oar-like tarsus with neatly arranged bristles provides the main propulsive force for swimming^15–18^. During the recovery stroke, the drag force generated by diving beetles is only 1/40 of the thrust force generated by diving beetles during the power stroke^3^, and the principle of drag-powered swimming allows the swimming efficiency of diving beetles to reach 84%^19^. Furthermore, the movements of the two hind legs are synchronous during forwarding or retreating. Synchronized-leg-swimming kinematics are more hydro-dynamically efficient than alternating-leg-swimming kinematics^20^. These features of diving beetles can potentially be used to design miniature underwater robots, and, as such, the swimming patterns of diving beetles have been analyzed from the perspective of robotics^1,16,21–25^. However, due to the lack of detailed data on the movement process of the diving beetle's hind legs and the hind legs were simplified as a two-link mechanism, the biomimetic robots retreated during the recovery stroke of swimming motion and the turning efficiency was low. At present, the underwater propulsion flexibility of most bionic underwater unmanned vehicles is not as good as that of diving beetles^26–29^. When multi-link mechanism moves underwater, the torques corresponding to different link attitudes are different due to the existence of the potential flow along the direction of the link^30^, the change characteristics of the joint angle of the hind legs may affect the energy consumption during this process. For the design of underwater robots that mimic diving beetles, the variable law of joint angles among the components of the hind legs requires further study. The present paper focuses on the joint angle changes of the hind leg components of diving beetles, and provides a kinematic model for the design of bionic unmanned underwater vehicles that mimic the diving beetle.

## Results

### Motion characteristics

**Figure 1:**
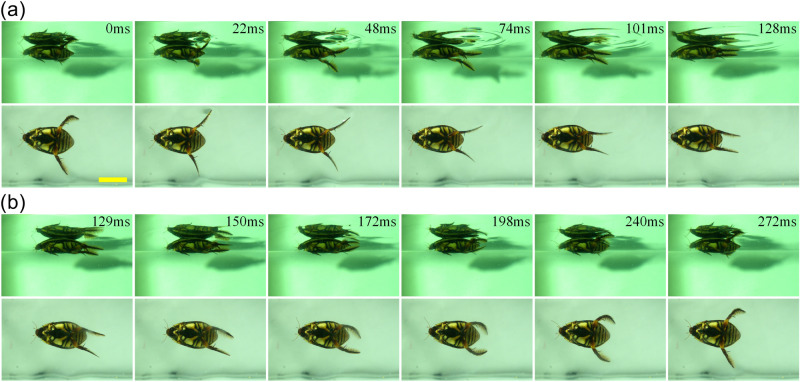
One forwarding cycle of a diving beetle: the period of motion is 272 ms, and includes **(a)** the power stroke and **(b)** the recovery stroke, the power stroke accounts for about 47% of the entire cycle, the scale bar = 20 mm.

As depicted in Fig. 1, the two hind legs were found to move symmetrically during forward motion, and the period of the motion cycle was 272 ms. The entire motion cycle was divided into the power stroke and the recovery stroke (the same for other swimming modes), and the former was found to account for about 47% of the cycle. In the early stage of the power stroke, each part of the hind legs stretched out, the hairs spread out and rotated with the tarsus, and finally pointed towards the back of the diving beetle. The result was that the cross-sectional areas against the water were increased, which contributed to the achievement of the maximized propulsion force. The tarsus rotated without any energy consumption^16^. The result of the tip of the femur moving at an angle to the ventral body surface found in this research is different from the findings of Hughes^31^. During the recovery stroke, the femur was found to move along the abdominal surface and the tarsus rotated to the original position (the tarsus was bent toward the inner edge, and the swimming hairs pointed to the tail). In addition, the hairs were found to fold up passively. These adaptations may reduce the resistance of the water during the recovery stroke to some degrees.

**Figure 2:**
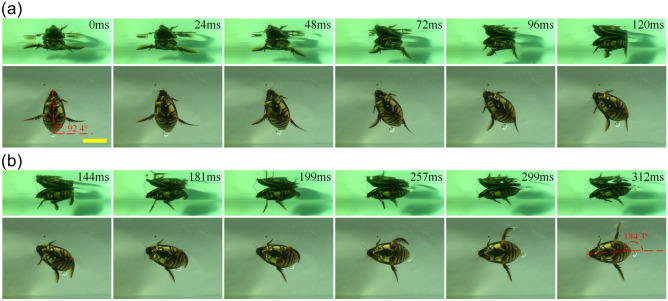
Time sequence diagram of a diving beetle for one right-turning cycle: the entire cycle of turning to the right is 312 ms, **(a)** the power stroke, **(b)** the recovery stroke, the scale bar = 20 mm, the top image of the diving beetle in the ventral view is the reflection in the water.

Conversely, the two hind legs were found to move unsymmetrically during the turning motion, and the time sequence diagram of the right-turning motion is illustrated in Fig. 2. The power stroke accounted for about 38% of the entire motion cycle. During the right-turning motion, the motion of the left hind leg was similar to that during forward motion, but the motion of the right hind leg was different. During the power stroke, the right hind leg was found to swing forward and the hairs folded up. During the recovery stroke, the right hind leg was found to swing backward with the leg stretched out and the hairs spread out. At the same time, the left hind leg swung forward with the hairs folded up. From the perspective of the forces acting on the hind legs, this may increase the stability of the turning motion of the diving beetle because the directions of the forces acting on the right and left hind legs are opposite. From Fig. 2, it can be found that the diving beetle had completed the turning motion, and this process only took one motion cycle. In other words, the diving beetle can turn flexibly and efficiently by controlling the asymmetric swing of its left and right legs, and the turning direction is opposite to that of the pushing hind leg.

**Figure 3:**
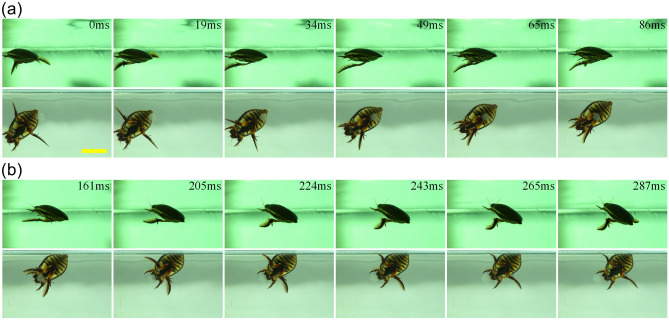
Time sequence diagram of a diving beetle for one retreating cycle: **(a)** the power stroke, which accounts for about 30% of the entire motion cycle; **(b)** the recovery stroke, the scale bar = 20 mm.

As presented in Fig. 3, the movements of the hind legs were found to be symmetrical during the retreating motion, and the power stroke accounted for about 30% of the entire motion cycle. At the start of the power stroke, the tibia and tarsus stretched out and quickly moved forward together with the femur. At the same time, the hairs spread out, and the tarsus produced a backward thrust by increasing the cross-sectional areas against the water. During the recovery stroke, the surface of the tarsus changed from being wide to narrow, and the hairs folded up. The tibia was found to swing backward along the abdominal wall, folded into the groove of the femur to reduce resistance, and then stretched out to prepare for the next cycle.

### Variation regularity of the joint angles of the hind legs

Only the movement joints on one leg were analyzed during the forward and retreating motion due to the symmetry of the movements of the two hind legs. However, the change characteristics of the joint angle of both hind legs during the turning motion were analyzed.

**Figure 4:**
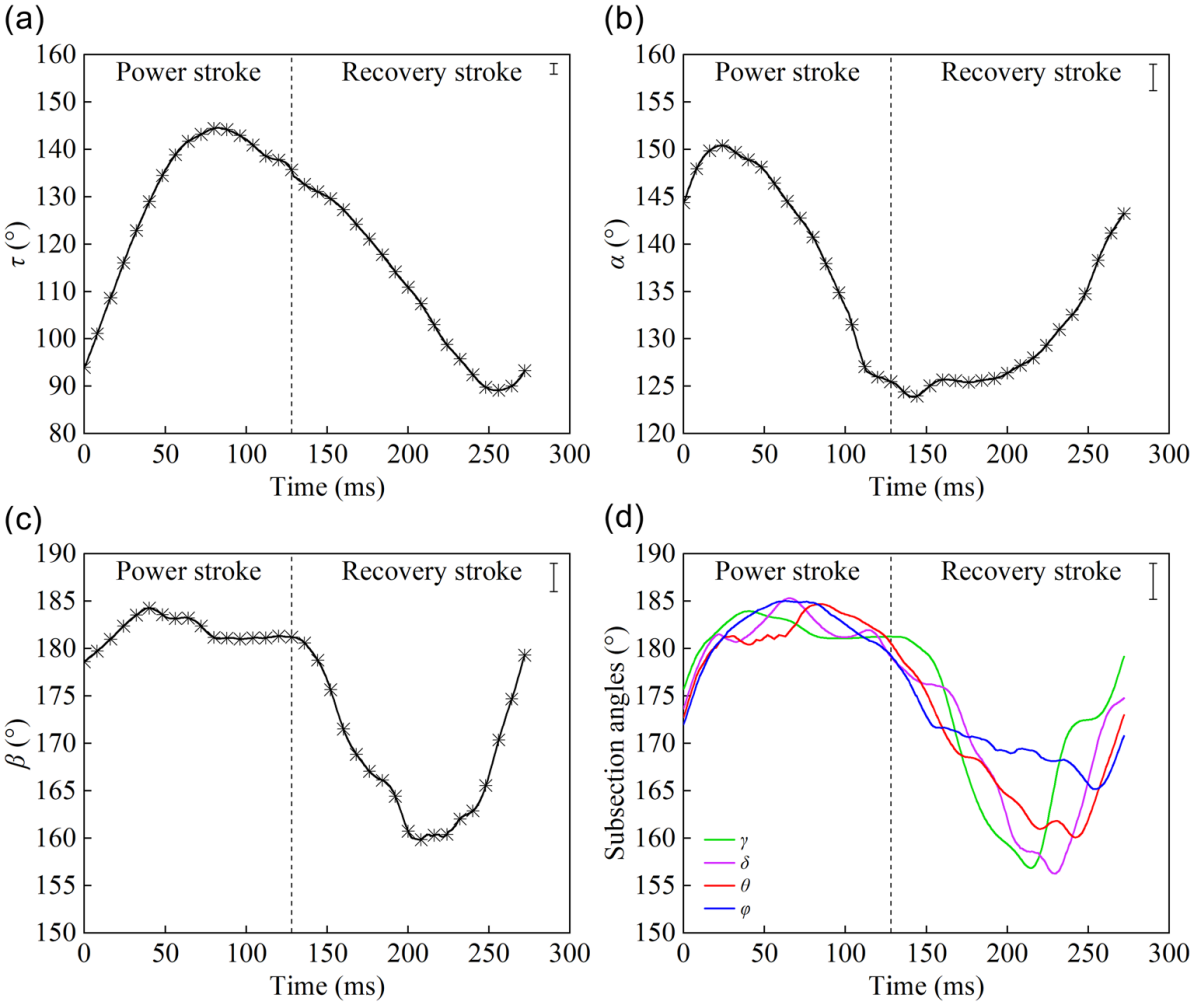
Diagram of the joint angle changes during forward motion: **(a)** the curve of angle *τ*; **(b)** the curve of angle *α*; **(c)** the curve of angle *β*; **(d)** curves of subsection angles (SIMI Motion 2007, www.simi.com, the carrier of the software is a disk).

As presented in Fig. 4, the change characteristics of the joint angles during forward motion were found to be as follows. During the power stroke, the femur swung backward and the angle *τ* increased greatly. At the end stage of the power stroke, the femur swung forwards slowly, and the angle *τ* began to decrease. During this part of the movement, the maximum joint angle *τ* was 145°. During the recovery stroke, the angle *τ* first decreased and then began to increase at the end. The swinging range of the femur was between 87° and 146° during the entire process (Fig. 4a). The joint angle *α* gradually increased at the start of the power stroke, for the tibia stretched outwards, then the tibia folded along the grooves of the femur, and the angle *α* decreased. The angle *α* was essentially unchanged at the end of the power stroke and early in the recovery stroke, and remained about 125°. The tibia then stretched outwards, causing the angle *α* to increase. The swinging range of the tibia was between 123° and 151° (Fig. 4b). By comparing the curves in Fig. 4c,d, it is evident that the trends of these curves are basically the same. The subsection angles remained in the range of 172°–185° during the power stroke. During the recovery stroke, with the retraction of the hind legs, the tarsus bent under the resistance of water, which caused these angles to decrease to minimal values. Afterward, at the end of the recovery stroke, these angles increased to prepare for the next cycle of movement.

**Figure 5:**
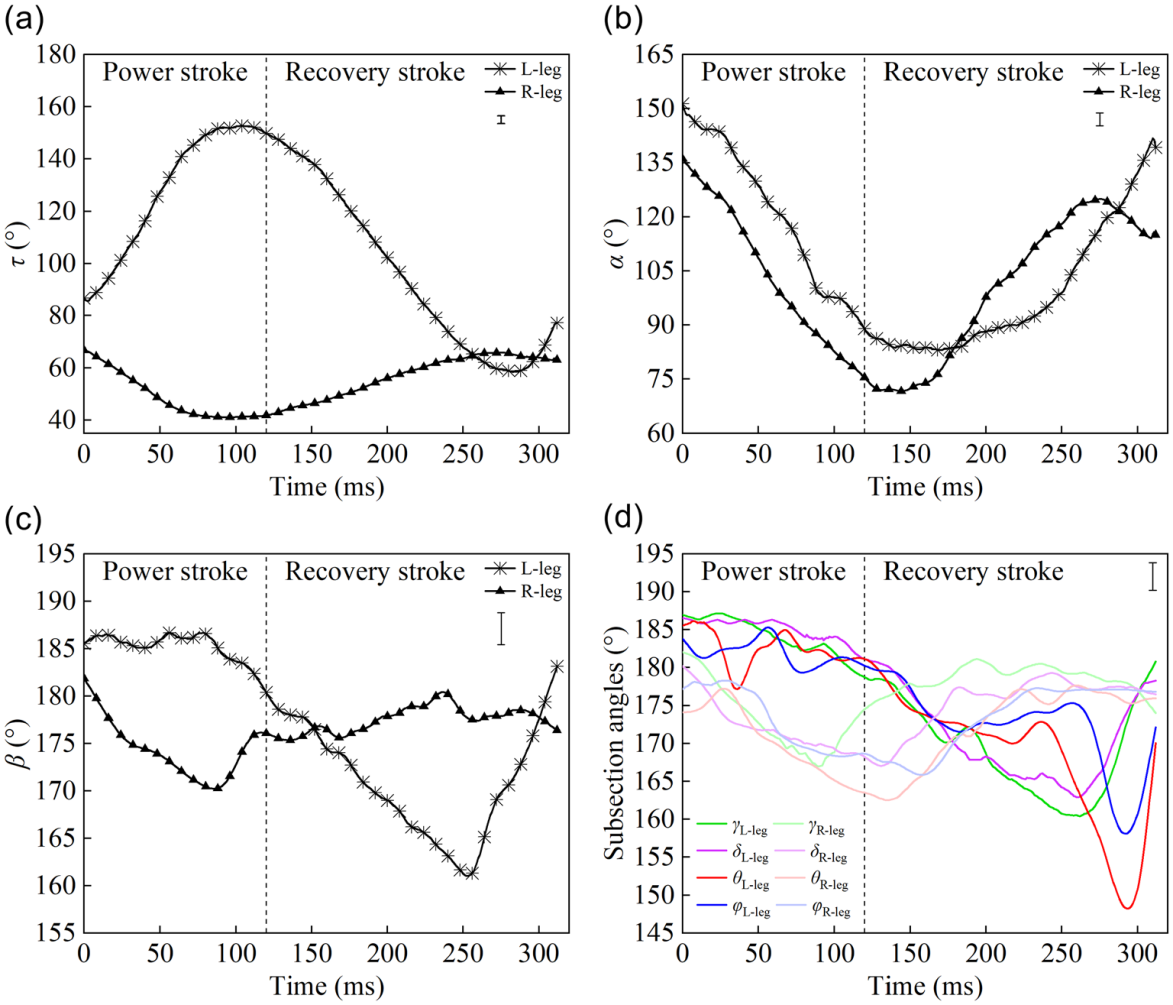
Curves of the joint angle changes with time during turning: **(a)** curves of angle *τ* of right and left hind legs; **(b)** curves of angle *α* of right and left hind legs; **(c)** curves of angle *β* of right and left hind legs; **(d)** the subsection angles of the left leg decreased slowly during the power stroke and increased rapidly at the end of the recovery stroke, while the subsection angles of the right leg remained relatively steady (SIMI Motion 2007, www.simi.com, the carrier of the software is a disk).

As presented in Fig. 5, during the turning motion, the joint angles (angle *τ*, angle *β*, and the subsection angles) of the left and right hind legs exhibited opposite trends, excluding the joint angle *α*. During the right-turning motion, the joint angle range of the right hind leg was less than that of the left leg. During the power stroke, the angle *τ* of the left hind leg increased rapidly while the angle *τ* of the right leg decreased slowly. During the recovery stroke, the angle *τ* of the left leg decreased greatly, and the angle *τ* of the right leg increased slightly (Fig. 5a). At the end of the recovery stroke, the two legs swung with the same angle of about 65° to prepare for the next movement cycle. Throughout the entire motion cycle, the femur of the left hind leg swung with an angle range of about 55°–155°, while the femur of the right hind leg swung with an angle range of about 40°–70°, which allows the right-turning movement to be completed quickly. The angle *α* of the left hind leg had the same tendency as the angle *α* of the right hind leg (Fig. 5b). During this movement cycle, the magnitude of the change in joint angle *α* was 70° ± 5°. During the power stroke, the joint angle *α* decreased because the tibia folded inwards along the groove of the femur. During the recovery stroke, the tarsus extended outward, leading to the increase of angle *α*. Figure 5c,d illustrate the variations of angle *β* and the subsection angles, and it is evident that the wave troughs of the joint angle curves of the left hind leg appeared during the recovery stroke, whereas those of the right hind leg appeared during both the recovery and power strokes. During the power stroke, the joint angles of the left hind leg were basically unchanged, while those of the right hind leg decreased rapidly. During the recovery stroke, the joint angles of the left hind leg first decreased and then increased, whereas those of the right hind leg first increased and then become relatively stable.

**Figure 6:**
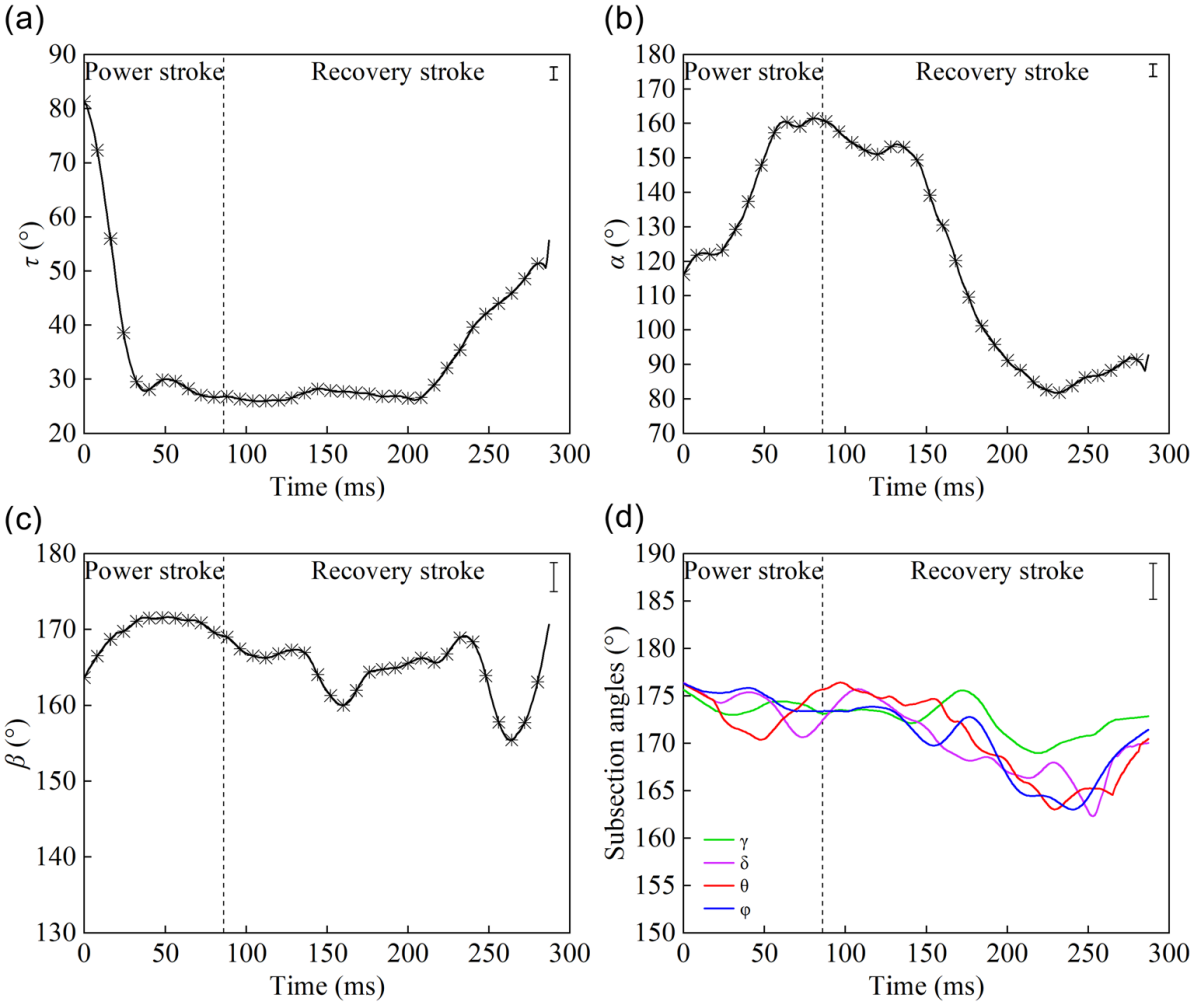
Curves of the joint angle changes with time during the retreating motion: **(a)** the curve of angle *τ*, which decreased rapidly during the power stroke and increased slowly during the recovery stroke; **(b)** the curve of angle *α*; **(c)** the curve of angle *β*; **(d)** curves of the subsection angles, the angle *β* and the subsection angles remained steady (SIMI Motion 2007, www.simi.com, the carrier of the software is a disk).

The angle *τ* decreased rapidly from 80° to 30° before 50 ms during the retreating motion due to the movement of the femurs (Fig. 6a). In the period from 50 to 200 ms, the femurs moved with a small amplitude. After 205 ms, the femurs swung backward, leading to an increase in angle *τ*. As presented in Fig. 6b, during the power stroke, the outwards extension of the tibia resulted in the increase of angle *α* from 116° to 165°. During the recovery stroke, angle *α* decreased rapidly because the tibia quickly returned to its original position. Finally, the tibia stretched outward to prepare for the next cycle. As presented in Fig. 6c, angle *β* at first remained relatively stable in the range of 160° to 175°, and then decreased and reached its minimum value because of the stretching and then bending of the tarsus. The subsection angles remained relatively stable in the range of 160° to 180°, as shown in Fig. 6d, and exhibited the lowest values during the bending of the tarsus throughout the recovery stroke.

### Movement velocity of diving beetles

**Figure 7:**
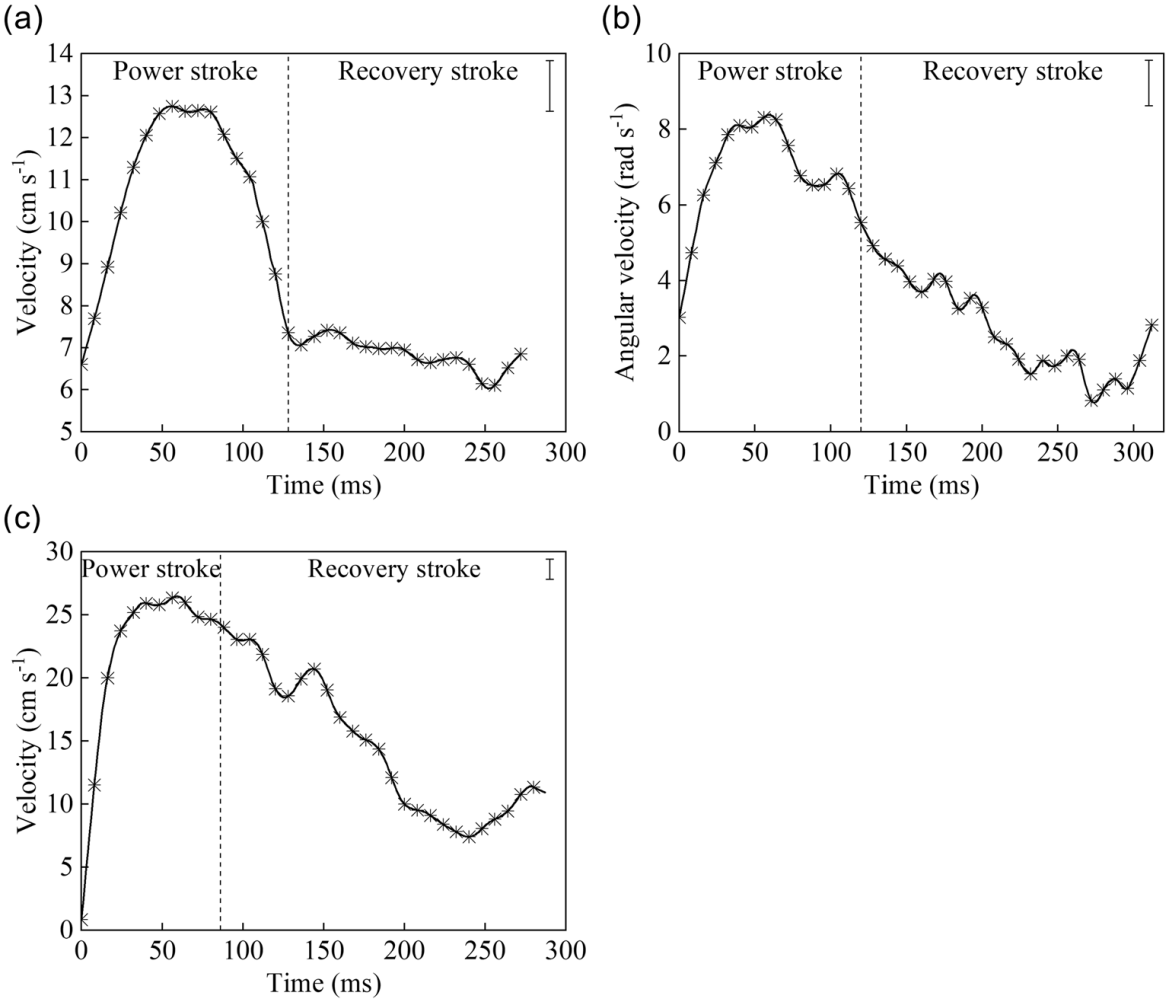
The speed of the diving beetle in the three movement modes: **(a)** velocity of the diving beetle during forward motion: the speed of diving beetle rapidly increased then rapidly decreased during the power stroke, and remained stable in the recovery stroke; **(b)** angular velocity of the diving beetle during turning motion: the angular velocity of diving beetle increased rapidly then decreased during the power stroke, and continued to decrease during the recovery stroke; **(c)** velocity of the diving beetle during retreating motion: the speed of the diving beetle increased rapidly during the power stroke and gradually decreased during the recovery stroke (SIMI Motion 2007, www.simi.com, the carrier of the software is a disk).

The speed of the diving beetle was obtained with the three-dimensional motion analysis software SIMI Motion. During the forward movement cycle (Fig. 7a), the maximum movement speed of the diving beetle was found to be as high as 12.9 cm/s, which occurred at about 60% of the power stroke, and the minimum movement speed was about 5.69 cm/s. The average acceleration in the first 50 ms was about 1.68 m/s^2^ and the average speed of this movement cycle was 8.74 cm/s. During the recovery stroke, the beetle moved forward at a relatively constant speed, which indicates that the structure of its legs can effectively reduce water resistance.

During the turning movement cycle, as presented in Fig. 7b, the maximum angular velocity of the diving beetle was found to be 8.3 rad/s, which occurred at about 56% of the power stroke. The angular velocity of the diving beetle then decreased to the minimum value of 0.83 rad/s. The average of the angular velocity was 4.4 rad/s.

As is presented in Fig. 7c, during the retreating movement cycle, the speed of the diving beetle was found to increase from 0 cm/s to 27.3 cm/s within 50 ms, which accounted for 58% of the power stroke. In the first 25 ms, the average acceleration was about 9.8 m/s^2^, which is equal to the acceleration of gravity. For reference, a car can accelerate to 100 km/h in just 2.83 s at this acceleration rate. Such a strong outbreak potential may be related to the diving beetle’s avoidance of natural enemies, when encountering danger, the beetle can quickly retreat to avoid injury.

### Kinematic Modeling of Diving Beetles

For one multi-link mechanism, it usually has rotating pair or moving pair, such as robot manipulator, which is the simplification of the human arm^32,33^. From the motion characteristics of the diving beetle’s hind leg, it is similar with the human arm. So the hind legs of a diving beetle can be represented by a multi-link mechanism (Supplementary Fig. S3). In order to accurately reflect the movement of the diving beetle’s hind leg, the principle of simplification is that all segments of the hind leg are simplified as equal-length links and all joints of the hind leg are simplified as rotating pairs. The coxa, trochanter, and femur are simplified as link I, the tibia is simplified as link II, and the tarsus is simplified as link IV. The links are connected by rotating pairs, which are respectively denoted as joint 1, joint 2, joint 3, and joint 4. From the analysis in the previous section, it can be determined that the joint between links II and IV has two degrees of freedom, and could therefore be regarded as two single-degree-of-freedom joints, namely joints 3 and 4. The rotation axes of joints 3 and 4 are perpendicular to each other, and the length of link III corresponding to joint 3 is 0^34^.

Supplementary Table S2 lists the Denavit-Hartenberg (D-H) parameters for the kinematic modeling of diving beetles^21,35,36^, in which *i* represents either joint 1, joint 2, joint 3, or joint 4 in Supplementary Fig. S3. The link’s rotation angle is represented by *λ*, and *a* is defined as the length of the link. Based on the obtained data (Supplementary Table S1), the lengths of the four links are respectively 10 mm, 5 mm, 0 mm, and 13 mm. The offset of the link is denoted by *d*, the numerical value of which is 0 because all coordinate systems are in one plane. The rotation amount about the common axis between one link and its neighbor is defined as *ψ*. (It is important to note that when link IV rotates counterclockwise around the coordinate axis *Z*_4_, it has a positive value; otherwise, it has a negative value. The unlabeled coordinate axes *Z*_1_, *Z*_2_, and *Z*_3_ are all perpendicular to the paper surface and point outwards.)

The general expression of the transformation matrix is as follows^34,37,38^, and the transformation matrices between different links are shown in Supplementary Equations:

1 $${}_{i}^{i - 1} T = \left[ {\begin{array}{*{20}c} {c\psi_{i} } & { - s\psi_{i} } & 0 & {a_{i - 1} } \\ {s\psi_{i} c\lambda_{i - 1} } & {c\psi_{i} c\lambda_{i - 1} } & { - s\lambda_{i - 1} } & { - s\lambda_{i - 1} d_{i} } \\ {s\psi_{i} s\lambda_{i - 1} } & {c\psi_{i} s\lambda_{i - 1} } & {c\lambda_{i - 1} } & {c\lambda_{i - 1} d_{i} } \\ 0 & 0 & 0 & 1 \\ \end{array} } \right]$$

Finally, the coordinates of joint 2 are

2 $$\left\{ \begin{gathered} P_{x2} = 10s\tau \hfill \\ P_{y2} = 10c\tau \hfill \\ P_{z2} = 0 \hfill \\ \end{gathered} \right.$$

and the coordinates of joint 3(4) are

3 $$\left\{ \begin{gathered} P_{x3(4)} = 5s(\alpha - \tau ) + 10s\tau \hfill \\ P_{y3(4)} = - 5c(\alpha - \tau ) + 10c\tau \hfill \\ P_{z3(4)} = 0 \hfill \\ \end{gathered} \right.$$

and the coordinates of the end of tarsus (point 5) are

4 $$\left\{ \begin{gathered} P_{x5} = - 13s(\alpha + \beta - \tau ) + 5s(\alpha - \tau ) + 10s\tau \hfill \\ P_{y5} = 13c(\alpha + \beta - \tau ) - 5c(\alpha - \tau ) + 10c\tau \hfill \\ P_{z5} = 0 \hfill \\ \end{gathered} \right.$$

where *s* represents the sine function and *c* represents the cosine function.

Finally, the motion trajectories of the hind legs of the diving beetle in different motion modes were obtained by inputting the trajectory equations and angle values of each joint into MATLAB software and calculating with it, and then drawing the trajectories at different points of time. As is presented in Supplementary Fig. S4, it is the motion trajectories of the left hind leg of a diving beetle during forward motion, which is symmetrical with that of the right hind leg. The motion trajectories of the hind leg were indicated by blue poly lines during the power stroke, and their color changed to red during the recovery stroke (the same for other motion modes). During the power stroke, the femur quickly swung towards the tail, angle τ increased by about 50°, the angle between tibia and tarsus was about 180° and the blue dotted line with arrows showed the trajectory of the end point of the tarsus. During the recovery stroke, a certain area was formed by the blue and red dotted line with arrows and what we could conclude is that the entire hind leg showed a clear contraction.

Similarly, the motion trajectories of the right and left hind leg of a diving beetle were also symmetrical during the retreating motion (showed in Supplementary Fig. S5). During the power stroke, the femur quickly swung towards the head, angle τ decreased by about 55°, the angle between the tibia and femur changed from 120° to 160° and the tibia and tarsus were roughly in line. During the recovery stroke, the entire hind leg of the diving beetle was curled up, the swing range of the femur was significantly smaller than that of during the power stroke. The shape of the area formed by the blue and red dotted line with arrows shows the characteristic of first thick and then thin, which shows that the diving beetle stretched its hind legs sufficiently to get a bigger propulsion force in the early stage of the retreating motion.

During the right-turning motion, the propulsive force was mainly provided by the left hind leg, and its trajectories were similar to that of the two hind legs in the forward motion, as is presented in Supplementary Fig. S6. The right hind leg was mainly used to control the direction of movement and adjust the balance of the body. During the power stroke, the femur swung towards the head, the rotation angle was about 25°, and the angle between the tibia and femur was reduced by about 70°. During the recovery stroke, the angle between the tibia and femur increased slowly, approaching the original value. The angle between the tibia and tarsus was about 180° during the whole movement, which was roughly in a straight line.

## Conclusion

Three common movement modes of the diving beetle were analyzed in this paper. All movement processes contain a power stroke and a recovery stroke. During the power stroke, diving beetles stretch their tibias and tarsi, and spread out the bristles to maximize the cross-sectional areas against the water to increase propulsion. During the recovery stroke, diving beetles reduce water resistance by rotating their tarsi 90° and folding the bristles.

The joint angle curves of the three movement modes were found to have similar trends (the turning motion only refers to the pushing leg). The changes of joint angle *β* and those of angles *γ*, *δ*, *θ*, *φ* were found to be basically the same for the three motion modes. During the power stroke, the ranges of the angles are 180° ± 5°. During the recovery stroke, the angles first decrease and then increase, and the ranges of the angle changes are 20° ± 5°.

In the three motion modes, the maximum velocity occurs at about 60% of the power stroke. The speed first increases quickly to a maximum value and then rapidly decreases. It takes only one motion cycle for the diving beetle to complete the turning motion. All of these indicate that the movement of the diving beetle is sufficiently flexible.

The kinematic model of the diving beetle was established and the motion trajectories of the hind legs of the diving beetle in different motion modes were also obtained, which may contribute to research on miniature unmanned underwater robots, especially their paddle propulsion systems.

## Materials and methods

### Diving beetles

*Cybister bengalensis*, a species of diving beetle, belongs to the class Insect, order Coleoptera, family Dytiscidae, and genus *Cybister* Curtis, and was the research subject of this study. All the diving beetles used in this study, which were obtained from Foshan City (Guangdong Province, China), were adults, and their average body length was 36.3 mm with a standard deviation of 0.67 mm. The body of the diving beetle comprises a head, chest, abdomen, two pairs of wings, three pairs of legs, and a pair of antennae. The pair of forelegs is used primarily for feeding and grabbing objects, the pair of middle legs can be used to stabilize the body during the recovery stroke while swimming, and the hind legs generate thrust for movement^39^. The main swimming organs of the diving beetle are their hind legs. Studies have shown that the hind legs are able to propel the beetle farther than the middle legs, and can generate a larger angular velocity than the middle legs^40^. The physical parameters of the hind legs of the diving beetle are displayed in Supplementary Table S1. Each hind leg consists of a coxa, trochanter, femur, tibia, and tarsus, and accounts for about 80% of the body length. The tarsus is the longest part of the hind leg, the length of the femur is approximately twice the length of the tibia, and the short tibia and long tarsus are adapted to high-speed swimming in open waters^41^. The femur is curved with a smooth outer surface, while the inner surface is distributed with pit structures. The surface of the tibia is non-smooth with some pits, in which burrs grow. The tarsus is comprised of 5 sub-sections that gradually narrow from the base to the end. The inner and outer edges of the tarsus have bristles with diameters of 5–20 μm (Supplementary Fig. S7), which is about 1/6 the width of human hair. These tarsal joints overlap one another on the surface and are anterior during retraction, thus increasing the rigidity of the structure^31^. The flattened femur, tibia, tarsus, and numerous swimming hairs assist with swimming^31,42^, and the change characteristics of the joint angle of the hind legs may affect the energy consumption of the diving beetle during this process^30^. When two articulated links rotate underwater, the torques corresponding to different link attitudes are different due to the existence of the potential flow along the direction of the link. As an aquatic creature, the diving beetle may adapt to the influence of this potential flow, and the kinematic model of its hind legs may improve the energy efficiency of paddle-driven robots to some extent. Therefore, the motions of the hind legs are of primary focus in the present study.

### Experimental environment

A motion capture system with two high-speed cameras was constructed, which was used to capture the motions of the hind legs of the diving beetles. The system is illustrated in Supplementary Fig. S1(a). The length and width of the transparent glass tank were 29 cm and 12 cm to ensure that the diving beetles could swim freely. An adjustable LED lamp was used to improve the visual brightness. The glass tank was covered with white cardboard for a bright visual background. Locomotion videos were captured by two synchronous high-speed digital cameras (Phantom® V711, Vision Research Inc., USA) from the bottom and side views of the transparent glass tank at a frame frequency of 1000 fps and a resolution of 1280 × 800. Before capturing the swimming of a diving beetle, a video of a calibration frame (Supplementary Fig. S1(b)) with 18 small balls was recorded for the analysis of motions. To improve the coordinate accuracy, at least 11 balls were placed in the visual field. After the calibration frame video was recorded, the calibration frame was removed from the water tank to record the movement of the diving beetle. Both the videos of the calibration frame and the movement of the diving beetle were imported together into SIMI Motion software for three-dimensional motion analysis. Even though the transparent glass tank is large enough for the diving beetle to swim freely, as a biological experiment object, it is still difficult to ensure the integrity of the period of the swimming motion, the straightness and flatness of the swimming route. We picked out three swimming videos that best meet the requirements from a large number of videos, which correspond to the three swimming modes (forwarding, turning, and retreating). To ensure the accuracy of analysis, we analyzed each video three times to calculate the average value of the data.

The main swimming modes of the diving beetle are forwarding, turning, and retreating, which were recorded in the experiments. Interestingly, the diving beetles could retreat without turning around, which was found to be realized by the forward swing of the hind legs, just like a car in reverse gear. In this paper, the feature points (white points in Supplementary Fig. S2) were traced by the shape and chromatism of the beetles’ joints.

## Supplementary Information

Supplementary Information 1.

Supplementary Information 2.

Supplementary Information 3.

Supplementary Information 4.

## Data Availability

All data generated or analysed during this study are included in this published article (and its Supplementary Information files).
