# Author Correction: Observation and analysis of diving beetle movements while swimming

**DOI:** 10.1038/s41598-021-97815-1

**Published:** 2021-09-03

**Authors:** Debo Qi, Chengchun Zhang, Jingwei He, Yongli Yue, Jing Wang, Dunhui Xiao

**Affiliations:** 1 Key Laboratory of Bionic Engineering (Ministry of Education), Jilin University, Room 218, Bionics Building, 5988# Renmin Street, Changchun, 130025 China; 2 State Key Laboratory of Automotive Simulation and Control, Jilin University, Room 218, Bionics Building, 5988# Renmin Street, Changchun, 130025 China; 3 Weihai Institute for Bionics, Jilin University, Weihai, 264402 China; 4 College of Physics, Jilin University, Changchun, 130012 China; 5 ZCCE, College of Engineering, Swansea University, Swansea, SA1 8EN UK

Correction to: *Scientific Reports* https://doi.org/10.1038/s41598-021-96158-1, published online 16 August 2021

The original version of this Article contained an error in the Acknowledgments section.

“The authors are grateful for the support of the National Key Research and Development Program of China (Grant No. 2016YFE0132900), the National Natural Science Foundation of China (Grant Nos. 51875243, 51575227), and the Science and Technology Development Program of Jilin Province (Grant No.20180101319JC).”

now reads:

“The authors are grateful for the support of the National Key Research and Development Program of China (Grant No. 2018YFA0703300), the National Natural Science Foundation of China (Grant Nos. 51875243, 51575227), and the Science and Technology Development Program of Jilin Province (Grant No.20180101319JC).”

The original Article has been corrected.

