# Supplementary material for: Observation and analysis of diving beetle movements while swimming
Source: Sci Rep. 2021 Aug 16;11:16581. doi: 10.1038/s41598-021-96158-1 (PMC8368022; doi:10.1038/s41598-021-96158-1)
Supplement: Supplementary file 2 — Supplementary Information 2. [file 41598_2021_96158_MOESM2_ESM.pdf]

Observation and analysis of diving beetle movements while swimming

Debo Qi[1], Chengchun Zhang[1,2,3,*], Jingwei He[1], Yongli Yue[1], Jing Wang[4], Dunhui Xiao[5]

[1]Key Laboratory of Bionic Engineering (Ministry of Education), Jilin University, Changchun 130025, China
[2]State Key Laboratory of Automotive Simulation and Control, Jilin University, Changchun 130025, China
[3]Weihai Institute for Bionics, Jilin University, Weihai 264402, China
[4]College of Physics, Jilin University, Changchun 130012, China
[5]ZCCE, College of Engineering, Swansea University, Swansea SA1 8EN, UK

***Corresponding author:**
Professor Chengchun Zhang
Key Laboratory of Bionic Engineering (Ministry of Education), Jilin University; State Key Laboratory of Automotive Simulation and Control, Jilin University; Weihai Institute for Bionics, Jilin University
E-mail: jluzcc@jlu.edu.cn
Telephone: (+86)0431-85095760-218
Room 218, Bionics Building, 5988# Renmin Street, Changchun 130025, China

The transformation matrix of link I is

$$ {}_1^0T = \begin{bmatrix} c\psi_1 & -s\psi_1 & 0 & 0 \\ s\psi_1 & c\psi_1 & 0 & 0 \\ 0 & 0 & 1 & 0 \\ 0 & 0 & 0 & 1 \end{bmatrix} = \begin{bmatrix} s\tau & -c\tau & 0 & 0 \\ c\tau & s\tau & 0 & 0 \\ 0 & 0 & 1 & 0 \\ 0 & 0 & 0 & 1 \end{bmatrix} \tag{1} $$

The transformation matrix between link I and link II is

$$ {}_2^1T = \begin{bmatrix} c\psi_2 & -s\psi_2 & 0 & 10 \\ s\psi_2 & c\psi_2 & 0 & 0 \\ 0 & 0 & 1 & 0 \\ 0 & 0 & 0 & 1 \end{bmatrix} = \begin{bmatrix} -c\alpha & s\alpha & 0 & 10 \\ -s\alpha & -c\alpha & 0 & 0 \\ 0 & 0 & 1 & 0 \\ 0 & 0 & 0 & 1 \end{bmatrix} \tag{2} $$

The transformation matrix between link II and link III is

$$ {}_3^2T = \begin{bmatrix} c\psi_3 & -s\psi_3 & 0 & 5 \\ s\psi_3 & c\psi_3 & 0 & 0 \\ 0 & 0 & 1 & 0 \\ 0 & 0 & 0 & 1 \end{bmatrix} = \begin{bmatrix} -s\beta & -c\beta & 0 & 5 \\ c\beta & -s\beta & 0 & 0 \\ 0 & 0 & 1 & 0 \\ 0 & 0 & 0 & 1 \end{bmatrix} \tag{3} $$

The transformation matrix between link III and link IV is

$$ {}_4^3T = \begin{bmatrix} c\psi_4 & -s\psi_4 & 0 & 0 \\ 0 & 0 & 1 & 0 \\ -s\psi_4 & -c\psi_4 & 0 & 0 \\ 0 & 0 & 0 & 1 \end{bmatrix} \tag{4} $$

The transformation matrix of coordinate system N relative to coordinate system 0 is

$$ {}_N^0T = {}_1^0T\,{}_2^1T\,{}_3^2T \cdots {}_N^{N-1}T \tag{5} $$

$$ {}_2^0T = {}_1^0T\,{}_2^1T = \begin{bmatrix} s(\alpha-\tau) & c(\alpha-\tau) & 0 & 10s\tau \\ -c(\alpha-\tau) & s(\alpha-\tau) & 0 & 10c\tau \\ 0 & 0 & 1 & 0 \\ 0 & 0 & 0 & 1 \end{bmatrix} \tag{6} $$

$$ {}_3^0T = {}_2^0T\,{}_3^2T = \begin{bmatrix} c(\alpha+\beta-\tau) & -s(\alpha+\beta-\tau) & 0 & 5s(\alpha-\tau)+10s\tau \\ s(\alpha+\beta-\tau) & c(\alpha+\beta-\tau) & 0 & -5c(\alpha-\tau)+10c\tau \\ 0 & 0 & 1 & 0 \\ 0 & 0 & 0 & 1 \end{bmatrix} \tag{7} $$

$$
{}^{0}_{4}T = {}^{0}_{3}T\,{}^{3}_{4}T = \begin{bmatrix} c(\alpha+\beta-\tau)c_4 & -c(\alpha+\beta-\tau)s_4 & -s(\alpha+\beta-\tau) & 5s(\alpha-\tau)+10s\tau \\ s(\alpha+\beta-\tau)c_4 & -s(\alpha+\beta-\tau)s_4 & c(\alpha+\beta-\tau) & -5c(\alpha-\tau)+10c\tau \\ -s_4 & -c_4 & 0 & 0 \\ 0 & 0 & 0 & 1 \end{bmatrix} \tag{8}
$$

where s represents the sine function and c represents the cosine function. Additionally, s_4 represents $\sin\psi_4$ and c_4 represents $\cos\psi_4$.
