# Supplementary material for: Observation and analysis of diving beetle movements while swimming
Source: Sci Rep. 2021 Aug 16;11:16581. doi: 10.1038/s41598-021-96158-1 (PMC8368022; doi:10.1038/s41598-021-96158-1)
Supplement: Supplementary file 3 — Supplementary Information 3. [file 41598_2021_96158_MOESM3_ESM.pdf]

Observation and analysis of diving beetle movements while swimming

Debo Qi[1], Chengchun Zhang[1,2,3,*], Jingwei He[1], Yongli Yue[1], Jing Wang[4], Dunhui Xiao[5]

[1]Key Laboratory of Bionic Engineering (Ministry of Education), Jilin University, Changchun 130025, China
[2]State Key Laboratory of Automotive Simulation and Control, Jilin University, Changchun 130025, China
[3]Weihai Institute for Bionics, Jilin University, Weihai 264402, China
[4]College of Physics, Jilin University, Changchun 130012, China
[5]ZCCE, College of Engineering, Swansea University, Swansea SA1 8EN, UK

***Corresponding author:**
Professor Chengchun Zhang
Key Laboratory of Bionic Engineering (Ministry of Education), Jilin University; State Key Laboratory of Automotive Simulation and Control, Jilin University; Weihai Institute for Bionics, Jilin University
E-mail: jluzcc@jlu.edu.cn
Telephone: (+86)0431-85095760-218
Room 218, Bionics Building, 5988# Renmin Street, Changchun 130025, China

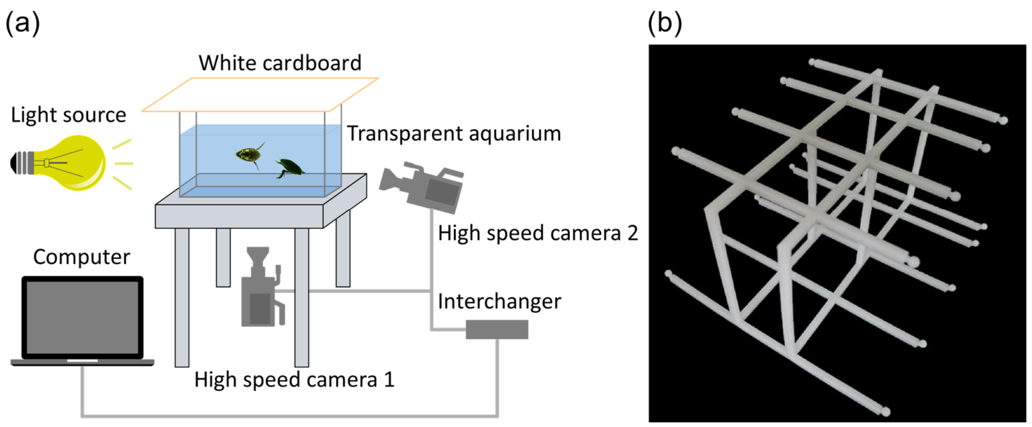

Supplementary Figure S1. Instruments and equipment used in the experiment: **(a)** the schematic of methods: a transparent aquarium was placed on the test platform and the surface of the test platform is transparent, an LED light was used to brighten the test environment, two high-speed cameras synchronously recorded from different angles, and the results were recorded by the connected computer; **(b)** the calibration frame for the marking of coordinate points: the calibration frame had two rows of small balls, and the radius of each ball was 1.75 mm, the colors of the balls were different to easily identify each coordinate point, the isometric 3D model of the calibration frame was printed by photosensitive resin material via 3D printing technology, the model of the calibration frame was moderately heated and softened to be corrected to prevent deformation caused by its small size during printing and transportation.

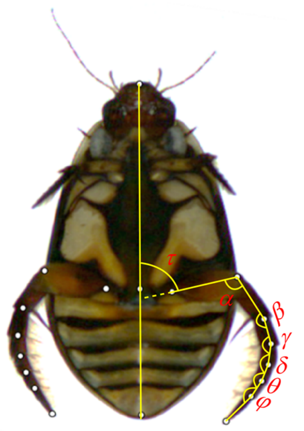

Supplementary Figure S2. The diagram of the tracked point locations and definitions of joint angles: the angle τ is the angle between the centerline of the body and the femur; the angle α is the angle between the femur and tibia; the angle β is the angle between the tibia and tarsus; angle γ, angle δ, angle θ, and angle φ are the angles on the tarsus, and are designated as "subsection angles" throughout the remainder of the paper.

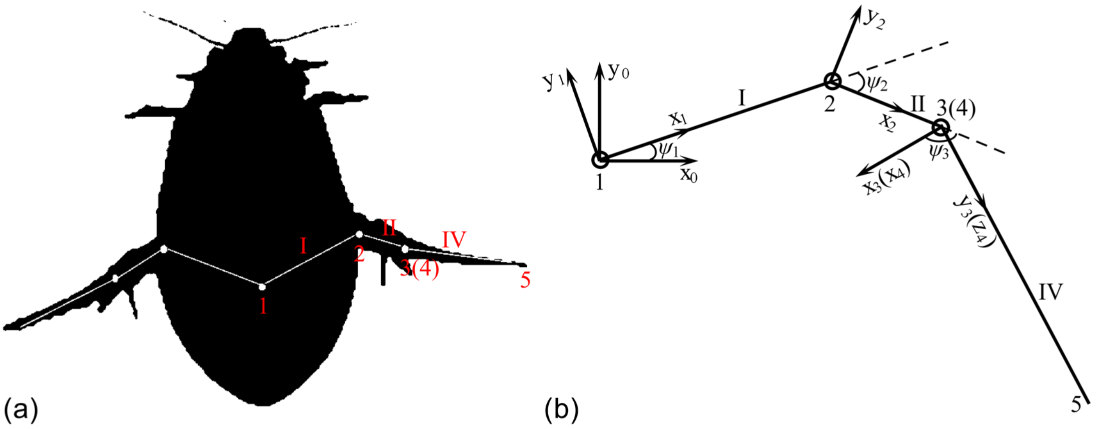

Supplementary Figure S3. The simplification of the hind legs of a diving beetle: **(a)** the simplified model of the diving beetle: the numbers 1, 2, and 3(4) represent joints, and I, II, and IV represent links; **(b)** the layout of the link-pole coordinate system of the right leg, where ψ represents the joint angle.

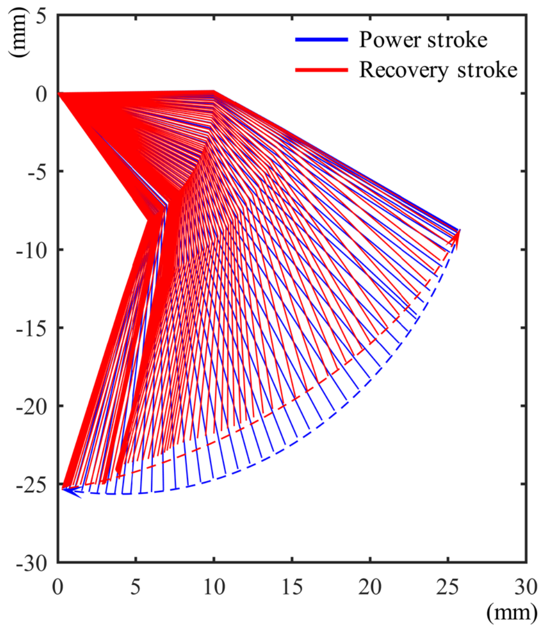

Supplementary Figure S4. The motion trajectories of the left hind leg of a diving beetle during forward motion. (MATLAB 2020 A, https://zbhrj1.jlu.edu.cn/download/matlab_2020a.html)

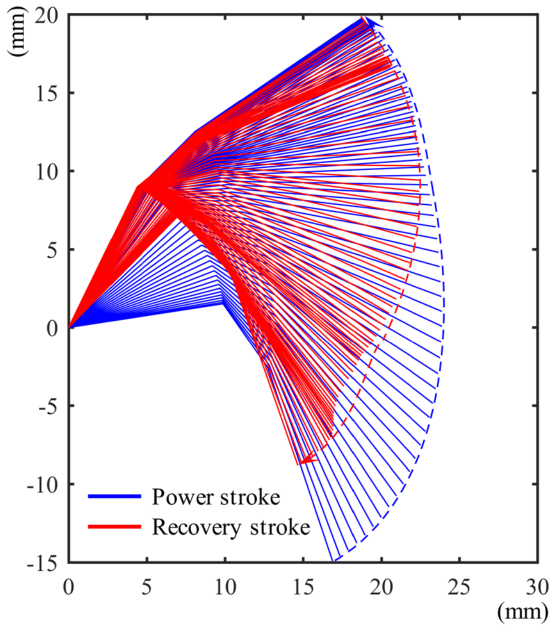

Supplementary Figure S5. The motion trajectories of the left hind leg of a diving beetle during retreating motion. (MATLAB 2020 A, https://zbhrj1.jlu.edu.cn/download/matlab_2020a.html)

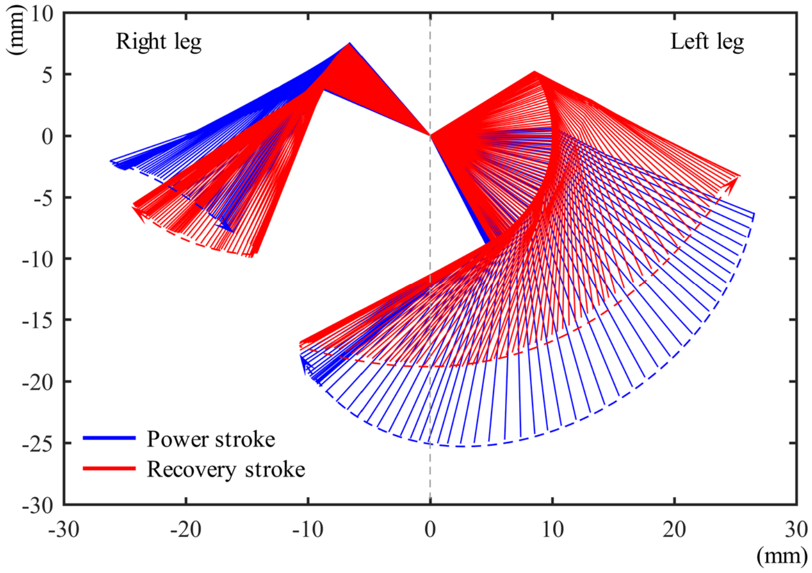

Supplementary Figure S6. The motion trajectories of the hind legs of a diving beetle during right-turning motion. (MATLAB 2020 A, https://zbhrj1.jlu.edu.cn/download/matlab_2020a.html)

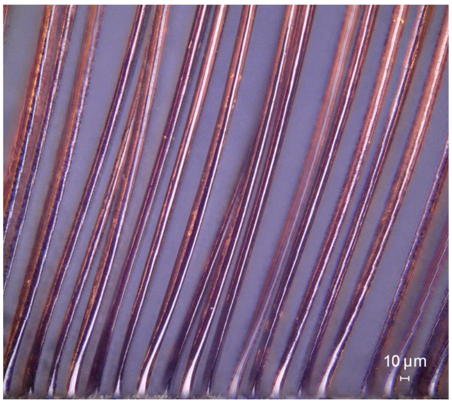

Supplementary Figure S7. Micrograph of swimming hairs taken with 200 times magnification
