# Supplementary material for: Observation and analysis of diving beetle movements while swimming
Source: Sci Rep. 2021 Aug 16;11:16581. doi: 10.1038/s41598-021-96158-1 (PMC8368022; doi:10.1038/s41598-021-96158-1)
Supplement: Supplementary file 4 — Supplementary Information 4. [file 41598_2021_96158_MOESM4_ESM.pdf]

Observation and analysis of diving beetle movements while swimming

Debo Qi[1], Chengchun Zhang[1,2,3,*], Jingwei He[1], Yongli Yue[1], Jing Wang[4], Dunhui Xiao[5]

[1]Key Laboratory of Bionic Engineering (Ministry of Education), Jilin University, Changchun 130025, China
[2]State Key Laboratory of Automotive Simulation and Control, Jilin University, Changchun 130025, China
[3]Weihai Institute for Bionics, Jilin University, Weihai 264402, China
[4]College of Physics, Jilin University, Changchun 130012, China
[5]ZCCE, College of Engineering, Swansea University, Swansea SA1 8EN, UK

***Corresponding author:**
Professor Chengchun Zhang
Key Laboratory of Bionic Engineering (Ministry of Education), Jilin University; State Key Laboratory of Automotive Simulation and Control, Jilin University; Weihai Institute for Bionics, Jilin University
E-mail: jluzcc@jlu.edu.cn
Telephone: (+86)0431-85095760-218
Room 218, Bionics Building, 5988# Renmin Street, Changchun 130025, China

Supplementary Table S1. Physical parameters of the diving beetles used in this study.

Number	Body (mm)	Femur (mm)	Tibia (mm)	Tarsus (mm)					
				Total	1	2	3	4	5
1	37	9.5	4.6	14.5	4	2.5	2	2	4
2	36	9	4.7	13.1	3.3	2.1	2	1.7	4
3	35.5	10	5.5	13.2	3.5	2	1.9	1.8	4
4	36	9.6	4.5	12.8	3	2	2	1.8	4
5	37	9.8	4.8	12.7	3	2	1.8	1.9	4

Supplementary Table S2. D-H (Denavit-Hartenberg) parameters.

Link *i*	λ_{i-1}	a_{i-1}(mm)	d_i(mm)	ψ_i
I	0	0	0	$\psi_1 = \pi/2 - \tau$
II	0	10	0	$\psi_2 = \alpha - \pi$
III	0	5	0	$\psi_3 = \beta - 3\pi/2$
IV	-90°	0	0	ψ_4
